# Supplementary material for: Photoinduced monooxygenation involving NAD(P)H-FAD sequential single-electron transfer
Source: Nat Commun. 2020 May 25;11:2600. doi: 10.1038/s41467-020-16450-y (PMC7248105; doi:10.1038/s41467-020-16450-y)
Supplement: Supplementary file 1 — Supplementary Information [file 41467_2020_16450_MOESM1_ESM.pdf]

Supplementary information

Photoinduced monooxygenation involving NAD(P)H-FAD sequential single-electron transfer

Ernst et al.

Supplementary Table 1: Michaelis-Menten kinetics of the photoreduction of PqsL, PqsL-O and PqsL-Q. Kinetic parameters were determined in three independent experiments. Experiments were conducted at room temperature with 10 µM PqsL, 200 µM 2-ABA (if indicated) and 1.25 mM NADH/NADPH in 30 mM phosphate/borate buffer, pH 8.3. Illumination was provided by a single LED (λ_{max}=466 nm, 20 nm full width at half maximum (FWHM), 1050 µmol photons s^{-1} m^{-2}).

		NADH	NADPH	NADH+2-ABA	NADPH+2-ABA
**PqsL wt**	K_M (µM)	310.9 ± 21.95	157.5 ± 18.87	31.40 ± 7.82	13.85 ± 2.291
	k_{cat} (s^{-1})	0.56 ± 0.016	0.605 ± 0.025	0.168 ± 0.009	0.181 ± 0.005
	k_{cat}/K_M (s^{-1} M^{-1})	$1.80 x 10^3$	$3.84 x 10^3$	$5.35 x 10^3$	$13.06 x 10^3$
**PqsL-O**	K_M (µM)	175.05 ± 12.55	127.8 ± 10.6	10.24 ± 1.481	7.338 ± 1.651
	k_{cat} (s^{-1})	0.342 ± 0.009	0.338 ± 0.0503	0.17 ± 0.005	0.147 ± 0.004
	k_{cat}/K_M (s^{-1} M^{-1})	$1.96 x 10^3$	$2.645 x 10^3$	$16.63 x 10^3$	$20.06 x 10^3$
**PqsL-Q**	K_M (µM)	353.5 ± 31.18	109.4 ± 12.84	35.03 ± 6.881	24.41 ± 3.264
	k_{cat} (s^{-1})	0.311 ± 0.012	0.262 ± 0.009	0.084 ± 0.004	0.082 ± 0.002
	k_{cat}/K_M (s^{-1} M^{-1})	$0.88 x 10^3$	$2.39 x 10^3$	$2.4 x 10^3$	$3.36 x 10^3$

Supplementary Table 2: Plasmids used in this study. Plasmids for the expression of *pqsL*-variants in *P. putida* and *E. coli*. pSE2 corresponds to the pHERD30T plasmid harboring wild type *pqsL* gene carrying sequences coding for an *N*-terminal 8x-histidine tag and a TEV protease cleavage site. Plasmids highlighted in bold were used for heterologous expression in *E. coli* and purification of the respective gene products. *In vivo* activities were determined in at least 2 biological replicates.

**Plasmid**	**Mutation in *pqsL***	***In vivo* activity rel. to wild type *pqsL* (%)**
pSE2-A	R41Y, I43R	27.34
pSE2-B	I43R	25.43
pSE2-C	G45R	27.34
pSE2-D	A164H	74.09
pSE2-E	G275R	24.65
pSE2-F	R41Y, I43R, G45R	34.76
pSE2-G	I43R, G45R	22.93
pSE2-H	A164H, G275R	26.87
pSE2-I	R41Y, I43R, G45R, A164H	12.49
pSE2-J	R41Y, I43R, G45R, G275R	12.56
pSE2-K	I43R, G45R, A164H	3.82
pSE2-L	I43R, G45R, G275R	1.66
pSE2-M	R41Y, I43R, G45R, A164H, G275R	2.56
pSE2-N	I43R, G45R, A164H, G275R	0.98
**pSE2-O**	**C105G**	**97.11**
**pSE2-P**	**G45R, C105G**	**72.35**
**pSE2-Q**	**R41Y, I43R, G45R, C105G**	**71.61**
**pSE2-R**	**R41Y, I43R, G45R, C105G, A164H**	**52.29**
pSE2-S	R41Y, I43R, G45R, C105G, A164H, G275R	20.36
pSE2-T	R178S	72.65
pSE2-U	R178S, G275R	93.50
pSE2-V	A164H, R178S, G275R	53.71
pSE2-W	R41Y, I43R, G45R, C105G, A164H, R178S, G275R	60.74
pSE2-X	R41Y, I43R,C105G	71.72
pSE2-Y	G45H,C105G	40.14
pSE2-Z	R41Y, I43R,G45H,C105G,A164H	37.06
pSE2-AA	Q36R	104.45
pSE2-AB	Q36R,A37Q	85.06
pSE2-AC	Q36R, G45R,C105G	15.86
pSE2-AD	Q36R,A37Q,G45R,C105G	29.26
pSE2-AE	G45R, C105G, A164H	12.75
pSE2-AF	P273G	66.64
**pSE2-AG**	**P273A**	**78.72**

Supplementary Table 3: Primers for site-directed mutagenesis, chromosomal integration of *pqsL* in *P. aeruginosa* and cloning of *6xhis-pqsL* in pHERD30T used in this study.

Purpose	Primer Name	Primer Sequence
Mutagenesis Q36R	pqsL-Q36R-F<br>pqsL-Q36R-R	TGGTAGTGGAACGGGCACGGCGCGAAC<br>GTTCGCGCCGTGCCCGTTCCACTACCA
Mutagenesis A37Q	pqsL-A37Q-F<br>pqsL-A37Q-R	TGGTAGTGGAACAGCAACGGCGCGAAC<br>GTTCGCGCCGTTGCTGTTCCACTACCA
Mutagenesis Q36R A37Q	pqsL-Q36R-A37Q-F<br>pqsL-Q36R-A37Q-R	TGGTAGTGGAACGGCAACGGCGCGAAC<br>GTTCGCGCCGTTGCCGTTCCACTACCA
Mutagenesis R41Y I43R	PqsL-R41Y-I43R-F<br>pqsL-R41Y-I43R-R	CACGGCGCGAATACGCGCGCAACGGCGCCGACCTGCTCAAG<br>GTCGGCGCCGTTGCGCGCGTATTCGCGCCGTGCCTGTTCCAC
Mutagenesis R41Y I43R G45R	pqsL-R41Y-I43R-G45R-F<br>pqsL-R41Y-I43R-G45R-R	GAATACGCGCGCAACCGCGCCGACCT<br>GCTTGAGCAGGTCGGCGCGGTTGCG
Mutagenesis I43R	pqsL-I43R-F<br>pqsL-I43R-R	GCGAACGCGCGCGCAACGGCGCCGACCTGCTCAAG<br>GTCGGCGCCGTTGCGCGCGCGTTCGCGCCGTG
Mutagenesis I43R G45R	pqsL-I43R-PqsL-G45R-F<br>pqsL-I43R-PqsL-G45R-R	GAACGCGCGCGCAACCGCGCCGACCT<br>CGGCTTGAGCAGGTCGGCGCGGTTGCG
Mutagenesis N44L	pqsL-N44L-F<br>pqsL-N44L-R	ACGCGCGATCCTCGGCGCCGACCTG<br>GTCGGCGCCGAGGATCGCGCGTTCGCG
Mutagenesis N44D	pqsL-N44D-F<br>pqsL-N44D-R	ACGCGCGATCGACGGCGCCGACCTG<br>GTCGGCGCCGTCGATCGCGCGTTCGCG
Mutagenesis G45R	pqsL-G45R-F<br>pqsL-G45R-R	CGCGATCAACCGCGCCGACCTGCTCAAGCCGGC<br>GGTCGGCGCGGTTGATCGCGCGTTCGCGCCG
Mutagenesis G45H	pqsL-G45H-F<br>pqsL-G45H-R	CGCGATCAACCACGCCGACCTGCTCAAGCCGGC<br>GGTCGGCGTGGTTGATCGCGCGTTCGCGCCG
Mutagenesis C105G	pqsL-C105G-F<br>pqsL-C105G-R	CGGCTATTTCATCCTCATGCCCGGCGAGTCG<br>TACCAGGCGGCGCAGCGACTCGCCGGGCATGAG
Mutagenesis A164H	pqsL-A164H-F<br>pqsL-A164H-R	CCGACGGTATCCACTCCTACGTGCGCCGCCGGCTG<br>GCACGTAGGAGTGGATACCGTCGGCTCCCACCACC
Mutagenesis R178S	pqsL-R178S-F<br>pqsL-R178S-R	GATATCGATGTGGAAAGCCGCCCCTACCC<br>GACGGGTAGGGGCGGCTTTCCACATC
Mutagenesis G275R	pqsL-G275R-F<br>pqsL-G275R-R	GCATCCCCATCCGCTACCTGAACCTGGACCGCTACTGG<br>GGTTCAGGTAGCGGATGGGGATGCCCTTGAAGCGC
Mutagenesis P273G	pqsL-P273G-F<br>pqsL-P273-R	GGCATCGGCATCGGCTACCTGAACC<br>GTAGCCGATGCCGATGCCCTTGAAGC
Mutagenesis P273A	pqsL-P273A-F<br>pqsL-P273A	GGCATCCGCATCGGCTACCTGAACC<br>GTAGCCGATGGCGATGCCCTTGAAGC
Genomic complementation of ΔpqsL	pqsL-gComp-5'<br>pqsL-gComp-3'	GGAACGACACGGAGACTCATCCATGACGGACAACCATATCGAT<br>CTCCGGCCGCGCTGGCGGGTTCAGCCGAGCGGCGC
Cloning 6xHis-PqsL in pHERD30T	pqsL-pSE2-F<br>pqsL-pSE2-R	TTAACTTTAAGAAGGAGATATACATACCCAATGGGCAGCAGCCATCAT<br>CCCAGATGCCGCTGGATCTGGCTCAGCCGAGCGGCGCCGG

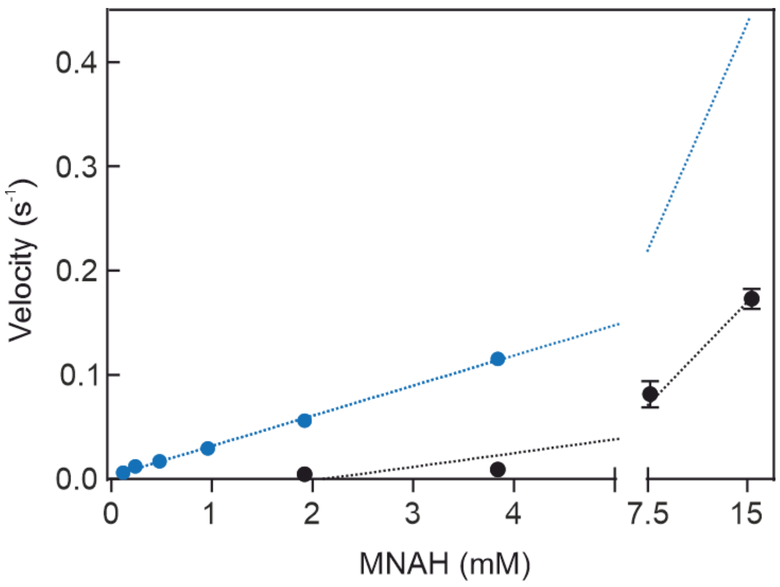

Supplementary Figure 1: Steady-state kinetics of FAD (blue) and PqsL (black) reactions with MNAH monitored by oxygen consumption. FAD concentration was kept constant at 10 µM and reactions were carried out at 25°C and atmospheric pressure. Bimolecular rates were determined from linear fits, assuming that flavin reoxidation rates were constant (k_{PqsL} = $1{\times}10^{-3}$ s^{-1} mM^{-1}, k_{FAD} = $3{\times}10^{-3}$ s^{-1} mM^{-1}). Error bars reflect standard deviations of n ≥ 3.

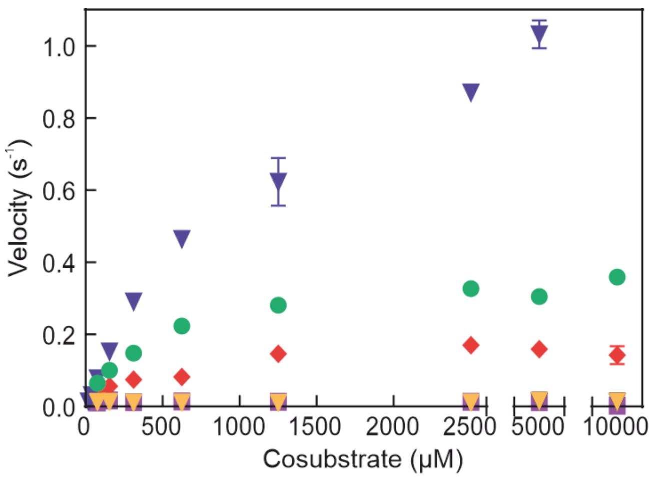

Supplementary Figure 2: Steady-state kinetics of PqsL photoreduction with various electron donors, as assayed by O_2 consumption (LED light source, λ_{max} = 466 nm, 20 nm full width at half maximum, 1050 µmol photons s^{-1} m^{-2}). Blue triangles: 1-methyl-1,4-dihydronicotinamide, red diamonds: ascorbic acid, green circles: EDTA, purple squares: triethylamine, orange triangles: L-glycine. Rates of the PqsL reactions were corrected for the contribution of residual free FAD (calculated based on the K_d of the PqsL-FAD complex). Error bars indicate SD of three identical measurements.

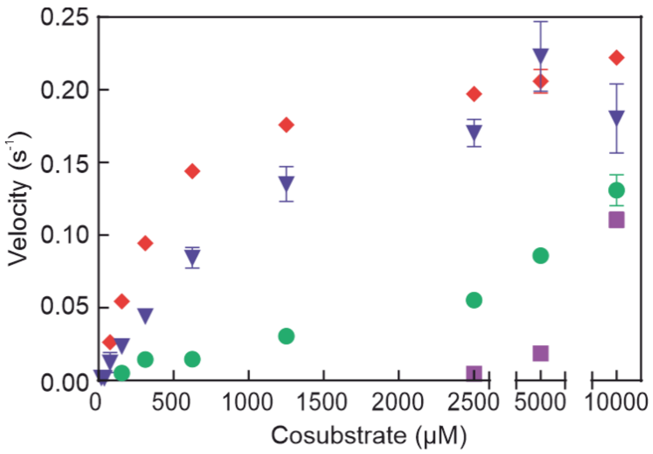

Supplementary Figure 3: Kinetics of FAD photoreduction with various electron donors, as assayed by O_2 consumption (LED light source, λ_{max} = 466 nm, 20 nm full width at half maximum, 1050 µmol photons s^{-1} m^{-2}). Blue triangles: 1-methyl-1,4-dihydronicotinamide, red diamonds: ascorbic acid, green circles: EDTA, purple squares: triethylamine. Error bars indicate SD of three identical measurements.

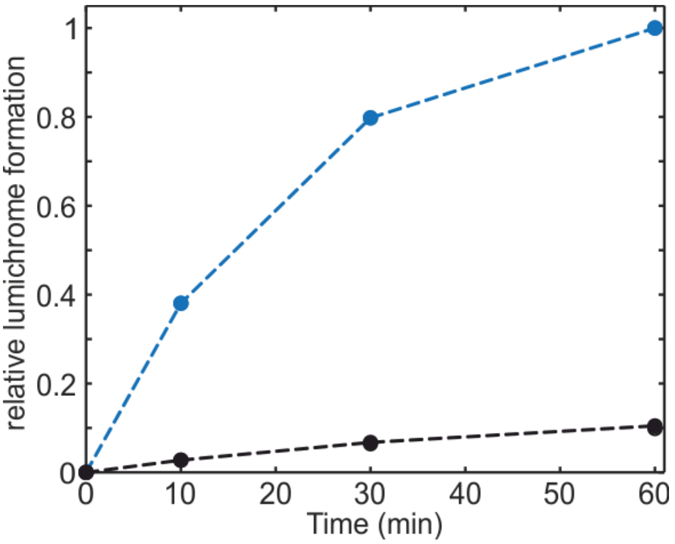

Supplementary Figure 4: Photodegradation of FAD. 500 µM of free FAD (blue) or PqsL (black) were illuminated in 20 mM tris-Cl, pH 8.5 with a blue light LED (λ_{max} = 466 nm, 20 nm full width at half maximum, 1050 µmol photons s^{-1} m^{-2}). Formation of lumichrome, the main product of FAD photodegradation, was assayed by HPLC. Free FAD was completely degraded after 60 min of illumination. One representative experiment shown.

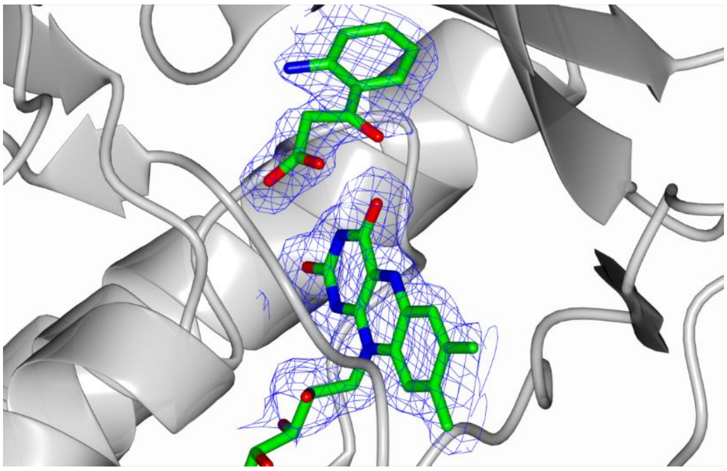

Supplementary Figure 5: Structure of PqsL with 2-ABA bound (**PDB ID: 6SW1)**. Blue grid represents electron densities for 2-ABA and the flavin ring.

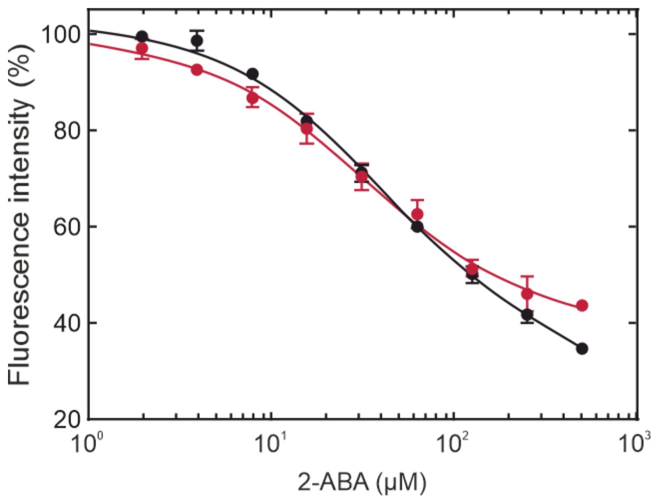

Supplementary Figure 6: Equilibrium titration of 2-ABA and PqsL. Changes of FAD fluorescence intensity upon 2-ABA titration to PqsL (black, K_d = 38.44 ± 3.98 µM) and PqsL-Q (red, K_d = 33.1 ± 4.7 µM). Excitation at 450 nm, 10 nm bandwidth; Emission measured at 520 nm, 10 nm bandwidth. Error bars indicate SD of three independent experiments.

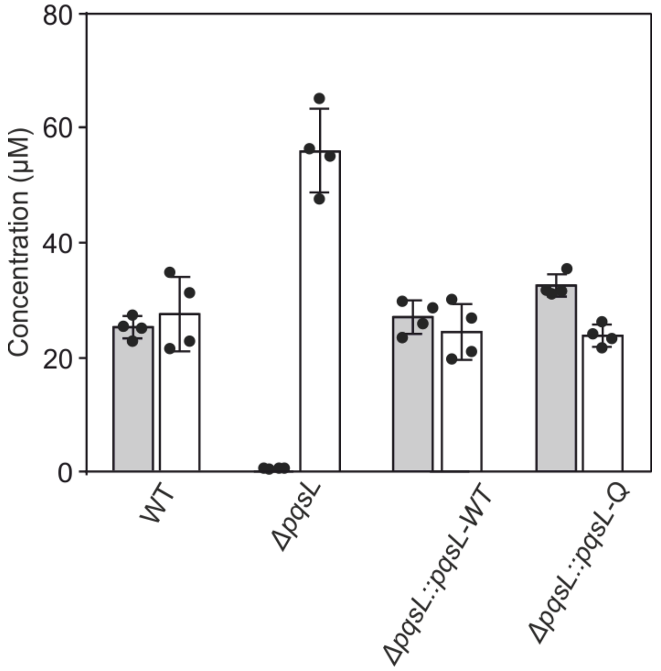

Supplementary Figure 7: Alkyl quinolone levels in different *Pseudomonas aeruginosa* strains. Depicted are concentrations of 2-heptyl-4-hydroxyquinoline-*N*-oxide (HQNO, gray bars) and the sum of 2-heptyl-3-hydroxy-4(1*H*)-quinolone + 2-heptyl - 4(1*H*)-quinolone (PQS, HHQ, white bars) in planktonic cultures. Error bars denote SD of four biological replicates.

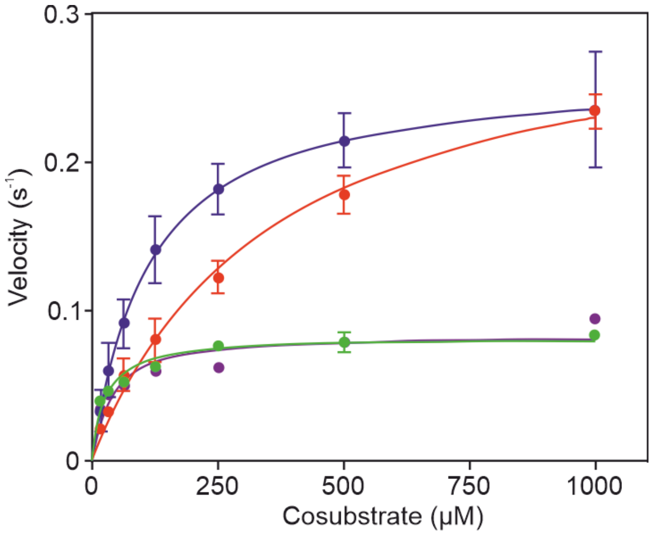

Supplementary Figure 8: Effects of NAD(P)H and 2-ABA on the photoreduction of PqsL-Q (LED light source). Blue: NADPH, red: NADH, green: NADPH + 200 µM 2-ABA, purple: NADH + 200 µM 2-ABA. Line traces reflect the respective Michaelis-Menten fits. Error bars indicate SD of three identical measurements.

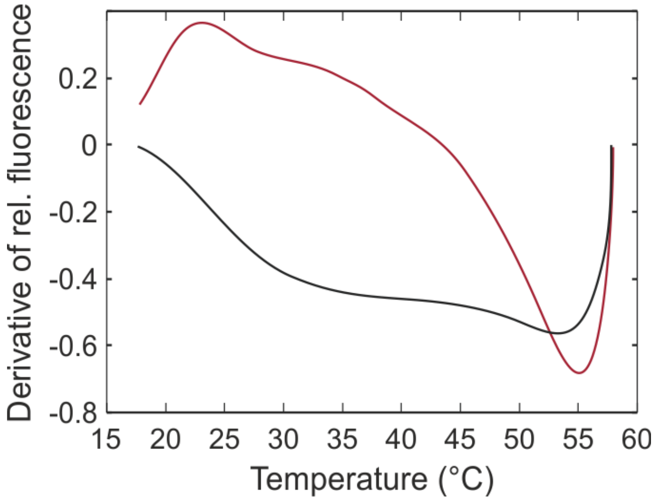

Supplementary Figure 9: Melting curve of PqsL (black) and PqsL-Q (red), the graph shows one representative experiment. 10 µM of the respective enzyme in 20 mM tris-Cl pH 8.5 were gradually heated in a spectrofluorometer and the changes in fluorescence intensity were recorded (λ_{ex} = 450 nm, λ_{em} = 520 nm, 10 nm bandwidth). Negative values in the melting curve indicate decrease in flavin fluorescence, which is indicative of FAD dissociation from the protein, e.g., due to protein unfolding or precipitation. Although melting temperatures of PqsL WT and PqsL-Q only differ by 1.4°C (WT 53.7°C vs 55.1°C), the gradual collapse of the PqsL WT holoprotein between 20 and 45°C does not occur in PqsL-Q, which was advantageous for spectroscopic procedures of the study and underlines the increased stability of the variant.

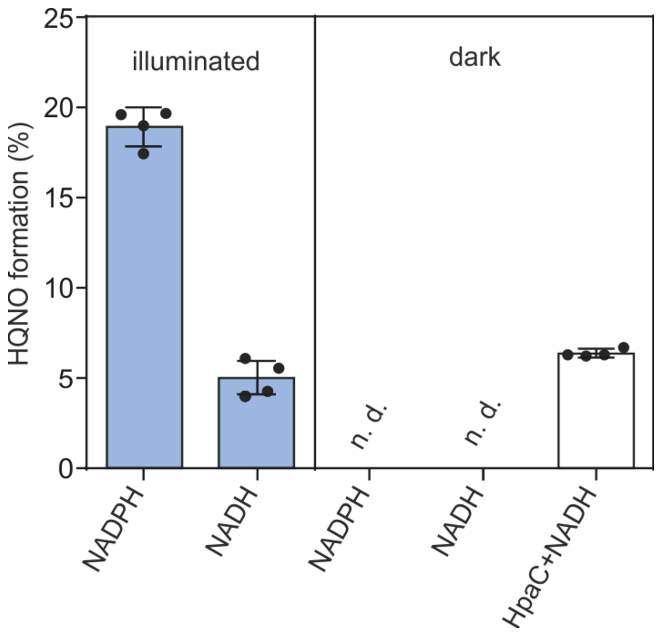

Supplementary Figure 10: Light-dependent product formation of PqsL-Q. Shown is the HQNO formation in the PqsL/PqsBC coupled enzyme assay. Error bars reflect SD of four independent measurements.

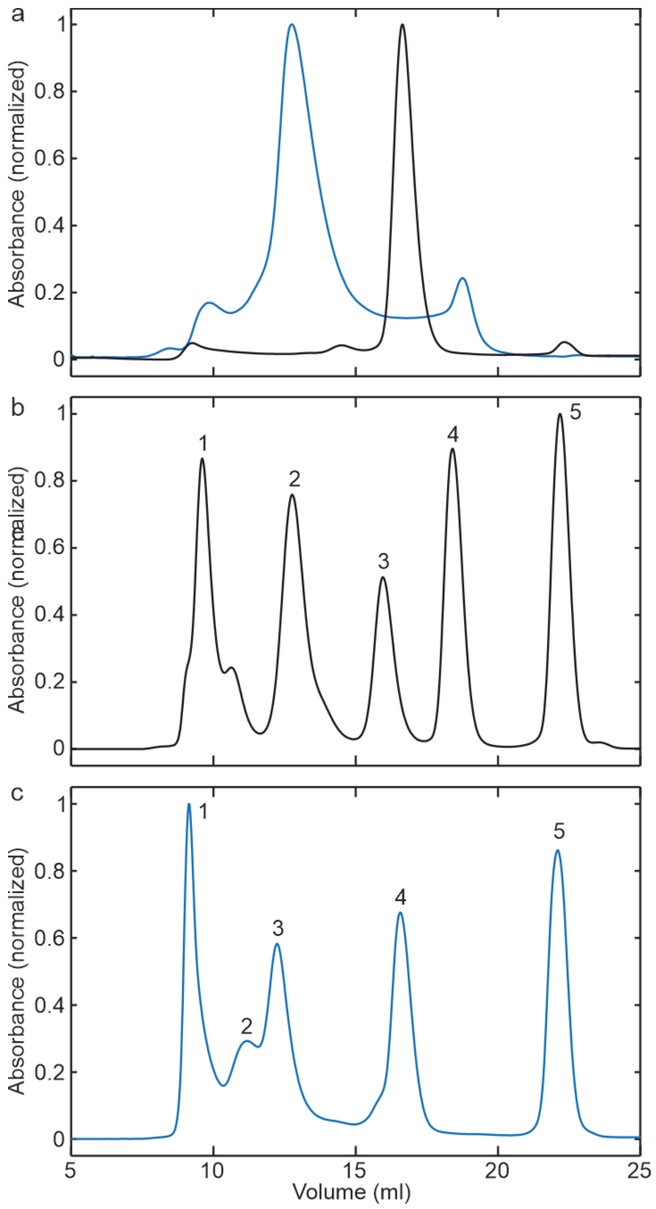

Supplementary Figure 11: Analytical gel filtration of PqsL. **a**, chromatograms of PqsL in 20 mM tris-Cl pH 8.5 (blue, retention volume 12.76 ml, calculated mass 99.1 kDa) and 20 mM tris-Cl, 150 mM NaCl pH 8.5 (black, retention volume 16.63 ml, calculated mass 34 kDa). **b**, chromatogram of the gel filtration standard (Bio-Rad, #1511901) in 20 mM tris-Cl, 150 mM NaCl, pH 8.5. 1: thyroglobulin (670 kDa); 2: γ-globulin (158 kDa); 3: ovalbumin (44 kDa); 4: myoglobin (17 kDa); 5: cobalamin (1.35 kDa) **c**, chromatogram of the gel filtration standard (Bio-Rad, #1511901) in 20 mM tris-HCl pH 8.5. Experiments were performed using a Superdex 200 Increase 10/300 GL with a constant flow rate of 0.6 ml min^{-1} at 12 °C.

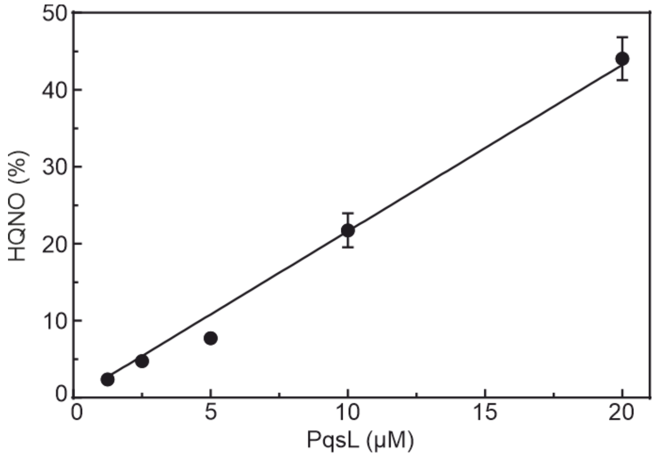

Supplementary Figure 12: HQNO formation in the coupled enzyme assay as function of PqsL concentration under constant illumination. Linearity suggests that no cooperative effects exist within the photoreduction within the covered concentration range. Error bars indicate SD of four independent measurements.

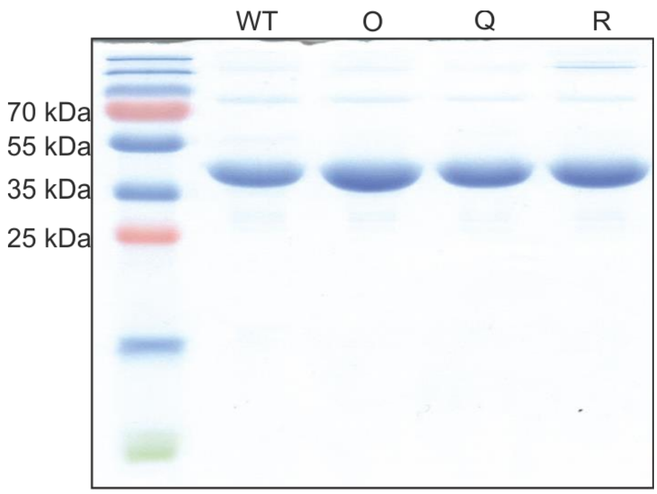

Supplementary Figure 13: SDS-PAGE of purified PqsL variants. 4 µg of protein loaded per lane, discontinuous 12.5% polyacrylamide gel.

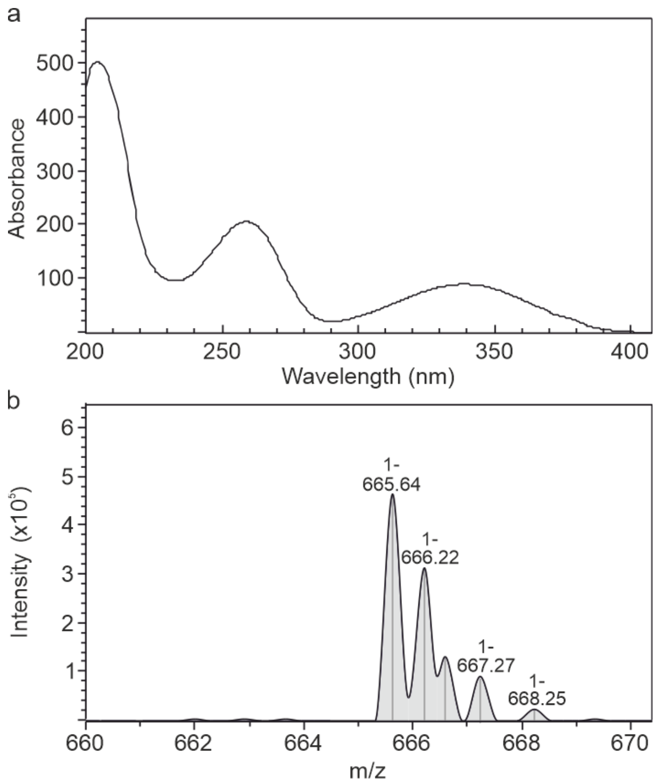

Figure 14: Representative UV/Vis-spectrum (**a**) of the synthesized reduced deuterated nicotinamide adenine dinucleotides in D_2O and the respective masses (**b**).
