# Supplementary material for: Photoinduced monooxygenation involving NAD(P)H-FAD sequential single-electron transfer
Source: Nat Commun. 2020 May 25;11:2600. doi: 10.1038/s41467-020-16450-y (PMC7248105; doi:10.1038/s41467-020-16450-y)
Supplement: Supplementary file 3 — Reporting Summary [file 41467_2020_16450_MOESM3_ESM.pdf]

nature research

Corresponding author(s): Steffen L. Drees

Last updated by author(s): Apr 9, 2020

# Reporting Summary

Nature Research wishes to improve the reproducibility of the work that we publish. This form provides structure for consistency and transparency in reporting. For further information on Nature Research policies, see Authors & Referees and the Editorial Policy Checklist.

## Statistics

For all statistical analyses, confirm that the following items are present in the figure legend, table legend, main text, or Methods section.

| n/a | Confirmed | |
|---|---|---|
| ☐ | ☒ | The exact sample size (*n*) for each experimental group/condition, given as a discrete number and unit of measurement |
| ☐ | ☒ | A statement on whether measurements were taken from distinct samples or whether the same sample was measured repeatedly |
| ☒ | ☐ | The statistical test(s) used AND whether they are one- or two-sided<br>*Only common tests should be described solely by name; describe more complex techniques in the Methods section.* |
| ☒ | ☐ | A description of all covariates tested |
| ☒ | ☐ | A description of any assumptions or corrections, such as tests of normality and adjustment for multiple comparisons |
| ☐ | ☒ | A full description of the statistical parameters including central tendency (e.g. means) or other basic estimates (e.g. regression coefficient) AND variation (e.g. standard deviation) or associated estimates of uncertainty (e.g. confidence intervals) |
| ☒ | ☐ | For null hypothesis testing, the test statistic (e.g. $F$, $t$, $r$) with confidence intervals, effect sizes, degrees of freedom and $P$ value noted<br>*Give P values as exact values whenever suitable.* |
| ☒ | ☐ | For Bayesian analysis, information on the choice of priors and Markov chain Monte Carlo settings |
| ☒ | ☐ | For hierarchical and complex designs, identification of the appropriate level for tests and full reporting of outcomes |
| ☒ | ☐ | Estimates of effect sizes (e.g. Cohen's $d$, Pearson's $r$), indicating how they were calculated |

*Our web collection on statistics for biologists contains articles on many of the points above.*

## Software and code

Policy information about availability of computer code

| | |
|---|---|
| Data collection | Standard software and/or established algorithms were used for X-ray and spectroscopic data collection. Software packages are referred to in the manuscript methods section. |
| Data analysis | Standard software and/or established algorithms were used for data analysis. Software packages are referred to in the manuscript methods section. |

For manuscripts utilizing custom algorithms or software that are central to the research but not yet described in published literature, software must be made available to editors/reviewers. We strongly encourage code deposition in a community repository (e.g. GitHub). See the Nature Research guidelines for submitting code & software for further information.

## Data

Policy information about availability of data

All manuscripts must include a data availability statement. This statement should provide the following information, where applicable:

- Accession codes, unique identifiers, or web links for publicly available datasets
- A list of figures that have associated raw data
- A description of any restrictions on data availability

Crystallization data and structure models are accessible through the pdb database structure repository (pdb codes 6SW1 and 6SW2).

# Field-specific reporting

Please select the one below that is the best fit for your research. If you are not sure, read the appropriate sections before making your selection.

☒ Life sciences ☐ Behavioural & social sciences ☐ Ecological, evolutionary & environmental sciences

# Life sciences study design

All studies must disclose on these points even when the disclosure is negative.

| | |
|---|---|
| Sample size | Sample sizes, as indicated in figure legends, were three or greater. When we chose to show a representative data set originating from a single measurement, the respective experiment was repeated at least three times. Data will be made available at reviewers' request. |
| Data exclusions | Data were only excluded if obvious mistakes, deviations from described protocols or instrument errors occured. |
| Replication | At least one completely independent replicate (i.e. new batch of protein/substrate/bacterial culture) was performed of every experiment. However, multiple partially independent experiments (multiple samples using the same protein/substrate batch) were performed, the exact numbers of which are given within the manuscript text. |
| Randomization | In microplate-based measurements, sample positions were randomized, in manual measurements, samples were measured in random order to minimize systematic error. |
| Blinding | The study does not contain experiments whose outcome is susceptible to human suggestion (such as placebo studies etc.). For non-linear fitting, starting values were calculated automatically based on common parameters to eliminate human bias. |

# Reporting for specific materials, systems and methods

We require information from authors about some types of materials, experimental systems and methods used in many studies. Here, indicate whether each material, system or method listed is relevant to your study. If you are not sure if a list item applies to your research, read the appropriate section before selecting a response.

## Materials & experimental systems

| n/a | Involved in the study |
|---|---|
| ☒ | ☐ Antibodies |
| ☒ | ☐ Eukaryotic cell lines |
| ☒ | ☐ Palaeontology |
| ☒ | ☐ Animals and other organisms |
| ☒ | ☐ Human research participants |
| ☒ | ☐ Clinical data |

## Methods

| n/a | Involved in the study |
|---|---|
| ☒ | ☐ ChIP-seq |
| ☒ | ☐ Flow cytometry |
| ☒ | ☐ MRI-based neuroimaging |
